# The potential role of glucose metabolism, lipid metabolism, and amino acid metabolism in the treatment of Parkinson's disease

**DOI:** 10.1111/cns.14411

**Published:** 2023-08-14

**Authors:** Hangzhen Li, Fancai Zeng, Cancan Huang, Qiqi Pu, Elizabeth Rosalind Thomas, Yan Chen, Xiang Li

**Affiliations:** ^1^ Department of Biochemistry and Molecular Biology, School of Basic Medical Science Southwest Medical University Luzhou China; ^2^ Department of Dermatology The Affiliated Hospital of Southwest Medical University Luzhou China; ^3^ Department of Microbiology NEIGRIHMS Shillong India

**Keywords:** amino acid metabolism, glucose metabolism, lipid metabolism, Parkinson's disease

## Abstract

**Purpose of Review:**

Parkinson's disease (PD) is a common neurodegenerative disease, which can cause progressive deterioration of motor function causing muscle stiffness, tremor, and bradykinesia. In this review, we hope to describe approaches that can improve the life of PD patients through modifications of energy metabolism.

**Recent Findings:**

The main pathological features of PD are the progressive loss of nigrostriatal dopaminergic neurons and the production of Lewy bodies. Abnormal aggregation of α‐synuclein (α‐Syn) leading to the formation of Lewy bodies is closely associated with neuronal dysfunction and degeneration. The main causes of PD are said to be mitochondrial damage, oxidative stress, inflammation, and abnormal protein aggregation. Presence of abnormal energy metabolism is another cause of PD. Many studies have found significant differences between neurodegenerative diseases and metabolic decompensation, which has become a biological hallmark of neurodegenerative diseases.

**Summary:**

In this review, we highlight the relationship between abnormal energy metabolism (Glucose metabolism, lipid metabolism, and amino acid metabolism) and PD. Improvement of key molecules in glucose metabolism, fat metabolism, and amino acid metabolism (e.g., glucose‐6‐phosphate dehydrogenase, triglycerides, and levodopa) might be potentially beneficial in PD. Some of these metabolic indicators may serve well during the diagnosis of PD. In addition, modulation of these metabolic pathways may be a potential target for the treatment and prevention of PD.

## Graphical abstract

Improvement of glucose metabolism, lipid metabolism, and amino acid metabolism is potentially useful for the treatment of Parkinson’s disease.

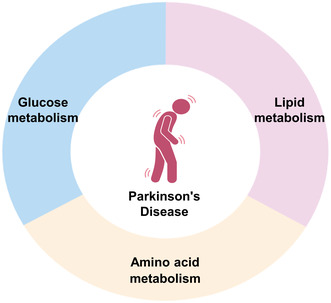

## 1 INTRODUCTION

Parkinson's disease (PD), also known as paralysis agitans, is a common neurological disorder of the middle‐aged and elderly people, most of whom present with symptoms of PD after the age of 60.^1^ It is currently believed that the pathogenesis of PD is mainly due to the loss of dopaminergic neurons from the substantia nigra (SN) of the midbrain, leading to the decreased activity of the dopamine transmitter system in the nigrostriatal area.^2^ In addition, Lewy bodies formed by abnormal aggregation of α‐synuclein (α‐Syn) are closely associated with the viability of dopaminergic neurons. At present, the detailed molecular mechanism of PD remains unmapped.

The main causes of PD are aging, environment factors, and genetic factors. These factors can induce apoptosis,^3^ autophagy dysfunction,^4^ mitochondrial dysfunction,^5^ and neuroinflammation,^6^ which ultimately causes neuronal death. Irrespective of the etiology of PD, disturbances in intracellular energy metabolism are observed during the development of PD.^7^, ^8^

It is well known that the main source of energy for any cell includes sugars, fats, and proteins. Previous studies have suggested that obesity can increase the risk of PD.^9^, ^10^ Most people with PD have been found to weigh less than normal and healthy individuals.^11^ Obviously, metabolic abnormalities of these factors (sugars, fats, and proteins) have been known to increase levels of cellular reactive oxygen species (ROS). The accumulation of ROS is said to be one of the main causes for induction of neurological diseases. Therefore, abnormalities in energy metabolism can induce changes in various indicators that can thereby behave as a molecular marker for PD.^8^ A previous study had reported that the incidence rate of PD was 1.23 times higher in individuals with metabolic syndrome when compared with individuals with no presentation of metabolic syndrome.^12^

The brain consumes around 25% of the body's glucose.^13^ Since the brain has no stored energy, glucose regularly crosses the blood–brain barrier to provide energy and aids in the synaptic transmission to neurons through glycolysis or mitochondrial oxidative phosphorylation. Evidence from postmortem tissues of PD patients indicates mitochondrial dysfunctions, compromised electron transport,^14^ and damaged tricarboxylic acid cycle (TCA).^15^ Aging is a major risk factor for developing PD. It has been shown that proteasomal and autophagic degradation of neurons is common during aging.^16^, ^17^, ^18^ Previous studies have reported that aging causes more vulnerability of the dopaminergic neurons when compared to the other brain cell.^19^ With aging, the decreased cellular metabolic activity triggers the accumulation of ROS, which leads to abnormal protein aggregation and finally results in damage to organelles.^20^ Eventually, it leads to the dysfunction of ubiquitin‐proteasome pathway and autophagy. Therefore, we suggest that it is the disruption of energy metabolism due to aging that promotes the accumulation of ROS, which inhibits autophagy and promotes apoptosis. Ultimately, it leads to neuronal loss and induces PD. Metabolic abnormalities accelerate brain aging.

High‐fat or/and high‐sugar diets can stimulate brain aging in mice and rats by triggering autophagy impairment,^21^ disturbance of Ca^2+^ homeostasis,^22^ and oxidative damage.^23^ Athauda and Foltynie had shown that insulin signaling normally regulates a variety of pathway in the brain, but it was found to be disrupted in individuals with PD.^24^ Metabolomics profiling of lipids in PD patients revealed that sebum may be identified as a potential biomarker for PD.^25^ A recent meta‐analysis indicates that the lipid serum triacylglycerols (TAGs) had a correlation with PD, and the levels of TAG in PD patients are significantly lower when compared to healthy individuals.^26^ Except for sugar and fat, amino acid metabolism also has a very important role in PD. Many amino acids, such as glutamate, γ‐aminobutyric acid (GABA), and glycine, are known to act as neurotransmitters used to regulate neuronal activity in a variety of neurological diseases. This is because the abnormal metabolism of these amino acids leads to neurotoxicity and neuronal death. In this review, we focus on the potential role of energy metabolism in the treatment of PD.

## 2 PARKINSON'S DISEASE AND GLUCOSE METABOLISM

Glucose is the main source of energy for the brain, and it is the main consumer of blood sugar in the resting state. Anandhan et al. have indicated that there is a relationship between alterations in glucose metabolism, oxidative stress, autophagy, and apoptosis caused by PD‐related risk factors.^27^ A recent study has reported that patients with diabetes mellitus have a higher risk of developing PD compared with healthy controls.^28^ This may be due to the disruption of glucose metabolism during diabetes, which is associated with dopamine dysfunction, especially in PD. For example, the level of dopamine is lower in a rat model of diabetes mellitus when compared to normal rat models.^29^ Low dopamine transporter binding in striatal and high cerebrospinal fluid α‐Syn (CSF) levels are found in patients with diabetes mellitus rather than in patients with PD.^28^ All these studies have illustrated that disorders in glucose metabolism of the body are significantly related to PD.

### 2.1 Parkinson's disease and glucose transporters

Glucose crosses the blood–brain barrier to enter the brain tissue; therefore, glucose transporters (GLUTs) play a very important role in brain energy metabolism. There are many different types of GLUTs, out of which, GLUT1, plays a key role in the functioning of the brain.^30^ GLUT3 is mainly expressed in neurons, while GLUT1 is mainly expressed in astrocytes.^31^ Puchades et al. had reported that the localization and densities of GLUT1 remain unaffected in a mouse model of PD,^32^ while Sarkar et al. had reported that GLUT1 is decreased in the striatum of a PD model of mice which was induced with (1‐Methyl‐4‐phenyl‐1,2,3,6‐tetrahydropyridine) MPTP.^33^ In addition, Burks et al. also had demonstrated a significant decrease of GLUT1 in the immunoreactive cells of the striatum after MPTP administration.^34^ Upregulating the expression of GLUT3 can inhibit the neurotoxicity induced in dopaminergic N27 cells which was treated with (1‐methyl‐4‐phenylpyridinium) MPP^+^ during in vitro research.^35^ Administration of STF‐31 (GLUT inhibitors) in dopaminergic cells can decrease paraquat toxicity which is used to induce PD models.^36^ Currently, there are limited reports on the potential use of GLUTs in the treatment and/or prevention of PD. The precise function of GLUTs in relation to PD is still unmapped. Recent studies have reported that antidiabetic medicines like exenatide may have a potential role in the treatment of PD.^37^, ^38^ The above‐mentioned findings have indicated that glucose transport and metabolism have a notable relation with PD.

### 2.2 Parkinson's disease and glycolysis

**FIGURE 1:**
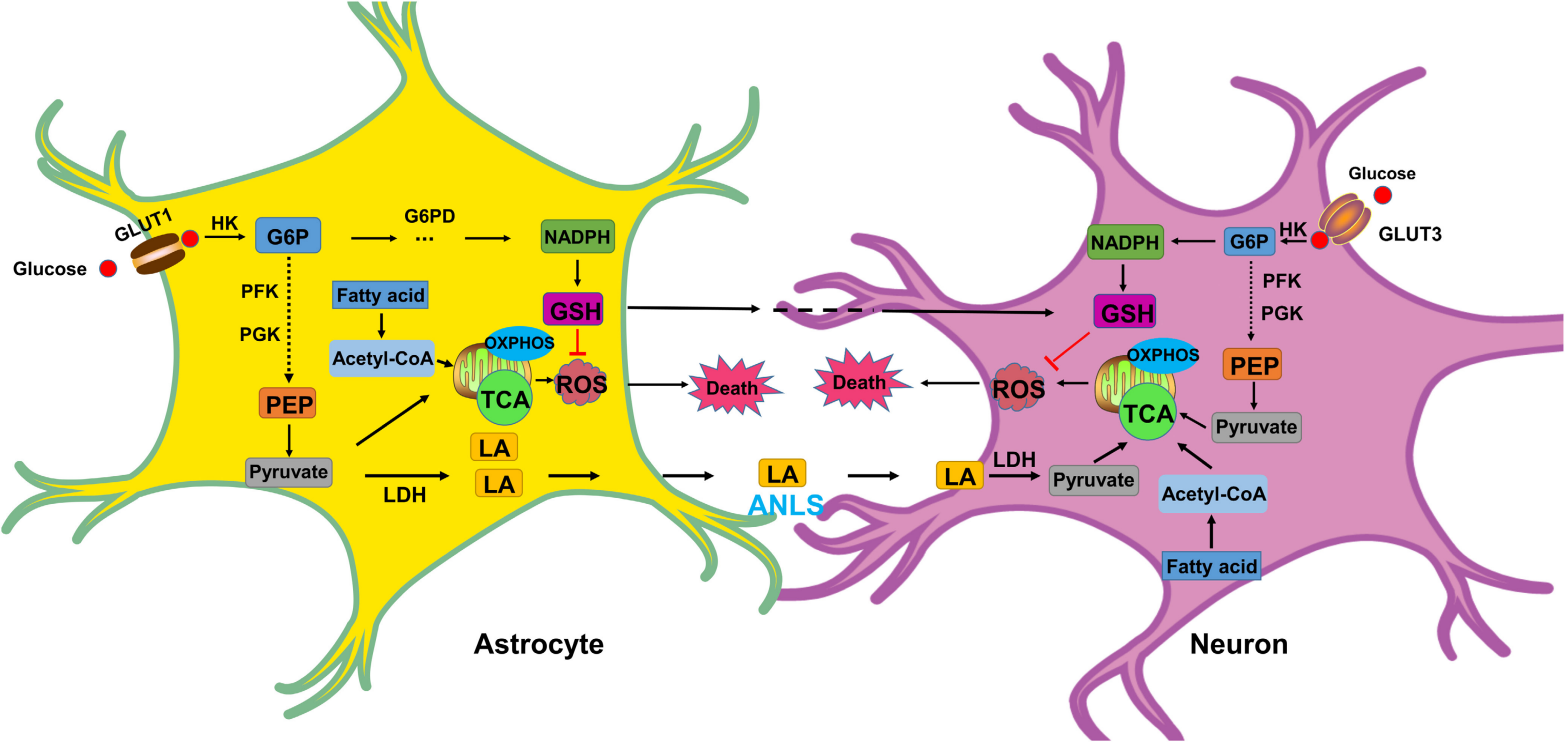
Overview of the glucose metabolism and fatty acids metabolism in neuronal and astrocytic compartments. Neurons and astrocytes can take up glucose respectively through GLUT3 and GLUT1. Glucose is phosphorylated by HK to generate G6P, which is subsequently routed in glycolysis and PPP. The end product of glycolysis is pyruvate. Acetyl‐CoA, derived from pyruvate or fatty acid oxidation, is channeled into the TCA cycle coupled with OXPHOS and ATP synthesis. Impaired mitochondrial energy metabolism leads to ROS accumulation which results in cell death. ATP generation is dependent on OXPHOS, while glucose metabolism is mainly directed toward the PPP to generate NADPH. NADPH is crucial for the GSH, which can reduce the accumulation of ROS. GSH can be shuttled to neurons to maintain redox homeostasis. LA enters the neuron by ANLS, and it can be converted to pyruvate by LDH, which provide energy for neurons. ANLS, astrocyte‐neuron‐lactate shuttle; G6P, glucose‐6‐phosphate; G6PD, glucose‐6‐phosphate dehydrogenase; GLUT1, glucose transporter 1; GLUT3, glucose transporter 3; GSH, glutathione; HK, hexokinase; LA, lactic acid; LDH, lactate dehydrogenase; OXPHOS, oxidative phosphorylation; PEP, phosphoenolpyruvate; PFK, phosphofructokinase; PGK, phosphoglycerate kinase; PPP, pentose phosphate pathway; ROS, reactive oxygen species; TCA, tricarboxylic acid cycle.

Disturbances in energy metabolism and reduced adenosine triphosphate (ATP) levels are common in PD.^39^ By and large, cortical glucose consumption is low during the early stages of PD.^40^, ^41^ A previous study has reported that brain aerobic glycolysis is necessary for synapse formation and growth;^42^ besides, enhanced glycolysis can alleviate PD.^43^ Although aerobic glycolysis is less efficient, it produces ATP faster than oxidative phosphorylation. During acute energy demand, glycolysis will temporarily exceed oxidative phosphorylation in neurons.^44^ Previous studies have reported that phosphoglycerate kinase 1 (PGK‐1) is a key metabolic enzyme for glycolysis, and deficiency of PGK‐1 is associated with PD.^45^, ^46^ Cai et al. had reported that terazosin can increase, ATP levels of brain and alleviate neuronal loss by enhancing the activity of PGK‐1. It can also increase dopamine levels and partially restore motor function in PD models of rats, mice, and flies.^43^ It has also been found that individuals who were administered terazosin or any of its related medicines have slower disease progression, fewer PD‐related complications, and less frequent PD diagnoses.^47^ Yang et al. proved that upregulation of leptin induced by NaHS can promote glycolysis and inhibit the loss of dopamine neurons in PD model of rats.^48^ Glucose can be catalyzed by hexokinase and transformed into glucose‐6‐phosphate (G6P). According to many in vivo and in vitro studies over the past decade, overexpression of hexokinase 2 (HK2) is found to alleviate the symptoms of PD by promoting glycolysis.^49^, ^50^ Meclizine activates the glycolytic enzyme phosphofructokinase (PFK), thus elevating glycolysis to resist PD.^51^ Doxazosin and alfuzosin can increase tyrosine hydroxylase level in a PD model of mice by activating glycolysis.^43^ Discontinuous energy metabolism and reduced ATP levels can increase the risk of PD.^43^ Promoting glycolysis may slow the progression of PD. Therefore, all these findings illustrate that glycolysis may play a key role in nervous system diseases (Figure 1).

### 2.3 Parkinson's disease and oxidative phosphorylation (OXPHOS)

The main site of oxidative phosphorylation (OXPHOS) is in the mitochondria of eukaryotic cells. ATP production is mainly dependent on OXPHOS pathway (consisting of five complexes I, II, III, IV, and V). Many genes such as Parkin, Pink1, DJ‐1, SNCA, and LRRK2 have been proved to be involved in PD. Mutation or deletion of these genes will lead to decrease in complex I activity and mitochondrial homeostasis, which results in cell death.^52^ Previous studies have demonstrated that mitochondrial dysfunction is an important cause of PD.^53^, ^54^ The susceptibility of DA neurons to mitochondrial dysfunction can be attributed to their high metabolic demand such as neurotransmitter release of high and long‐branched axon.^55^, ^56^ OXPHOS is the primary approach by which dopaminergic neurons obtain energy. This causes a sustained stimulation of mitochondrial OXPHOS, which increases oxidative damage to mitochondria.^57^ Current environmental toxicants, such as 1‐methyl‐4‐phenyl‐1,2,3,6‐tetrahydropyridine and Rotenone, induces PD by damaging the mitochondrial electron transport chain (ETC), resulting in impaired oxidative phosphorylation.^58^, ^59^ It is found that mtDNA mutations in SN neurons of patients with PD and aged humans are accompanied by a deficiency in OXPHOS. This results in the accumulation of ROS, which ultimately leads to mitochondrial dysfunction causing PD.^60^, ^61^ It is obvious that the accumulation of ROS induces neuronal apoptosis and autophagy inhibition in PD.^62^ The mitochondrial permeability transition (MPT) has a critical role in PD.^63^ Previous studies have reported that when MPT occurs, the mitochondrial membrane potential collapses, leading to disrupted oxidative phosphorylation and cell death.^64^, ^65^ Visch et al. have shown that influx of Ca^2+^ in mitochondria can promote OXPHOS and ATP production by regulating mitochondrial dehydrogenase enzymes.^66^ Imbalance of Ca^2+^ influx in the mitochondria is one of the causes of PD.^67^ Different types of cells respond to stress by enhancing oxidative phosphorylation such as inflammation, starvation,^68^, ^69^ and ionic imbalance.^70^ This generates large amounts of ROS. Therefore, we can suggest that OXPHOS dysfunction‐induced impairment of energy metabolism is one of the major reasons for PD by influencing MPT and Ca^2+^ signaling, which eventually leads to neuronal apoptosis or autophagy inhibition.

### 2.4 Parkinson's disease and gluconeogenesis

Gluconeogenesis is the conversion of a variety of non‐sugar substances into glucose or glycogen. The major sites of gluconeogenesis are the liver and kidneys. Mazzio et al. proved that intermediates of gluconeogenesis, such as pyruvate, malate, and phosphoenolpyruvate, have neuroprotective effects against MPP^+^‐induced neurotoxicity. These intermediates promote anaerobic substrate‐level phosphorylation, thereby promoting glycolysis.^71^ In addition, Kim et al. reported that MPTP triggered the expression of glycolysis and gluconeogenesis‐related proteins in mice.^72^ At present, there are few reports on the relationship between gluconeogenesis and PD. According to the above data, we suggest that the intermediate products of gluconeogenesis have a regulatory role in PD. This regulatory role is achieved by triggering glycolysis which in turn triggers oxidative phosphorylation.

### 2.5 Parkinson's disease and pentose phosphate pathway

The pentose phosphate pathway (PPP) is one of the mechanisms for oxidative breakdown of glucose. Previous studies have reported that the level of glucose‐6‐phosphate dehydrogenase (G6PD), a key enzyme of the PPP, is low during the early stages of PD.^73^ PPP is the main source of nicotinamide adenine dinucleotide phosphate (NADPH) in cells when compared to normal energy supply.^74^ Mejías et al. reported that overexpression of G6PD in DA neurons can alleviate the neurotoxicity induced by MPTP in mice.^75^ Therefore, the most important function of PPP in neurons is to regulate redox homeostasis by consuming glucose.^76^ This indicates that elevated NADPH levels resist PD by reducing the accumulation of ROS. However, Tu et al. reported that the expression of G6PD is increased in all the four different in vivo PD models.^77^ In addition, Lei et al. demonstrated that overexpression of G6PD using paraquat promotes toxicity, while inhibition of G6PD using 6‐aminonicotinamide alleviates mitochondrial functional impairment induced by paraquat in DA neurons.^78^ These findings suggest that excessive NADPH aggravates the neurotoxicity effect of paraquat, lipopolysaccharide, and MPTP in the mitochondria. It is the presence of NADPH during the oxidative cycle of these toxicants that promotes mitochondrial death. Therefore, the relationship between G6PD and PD is still controversial.^79^, ^80^ From the above‐mentioned evidences, four major reasons can be found accountable for this phenomenon. (1) Drug‐induced PD and idiopathic PD may have a difference in pathogenic mechanisms. (2) The role of G6PD may be different during different stages of PD such as the early stage of PD and the late stage of PD. (3) Influenced by different factors such as heredity, environment, and aging, the activity of G6PD during PD may be different. (4) G6PD can influence the neurons or/and astrocytes during PD because astrocytes provide both energy and nutritional support for neurons. Ohno et al. suggested that the PPP activity in astrocytes is five to seven times higher than neurons.^81^ In addition, a previous study has reported that the maintenance of neuronal redox homeostasis is largely dependent on the supply of reduced glutathione (GSH) by astrocytes because GSH or its metabolites can be shuttled to neurons.^82^ Therefore, all the above evidences indicate that the PPP is closely associated with PD. This association is made possible by the involvement of PPP in neuronal or/and astrocyte metabolism^75^, ^77^, ^83^ (Figure 1).

## 3 PARKINSON'S DISEASE AND LIPID METABOLISM

Some of the most common lipids that are consumed by humans include triacylglycerols (TAGs), cholesterols, and phospholipids.^84^ Lipids in the brain play an important component role in the structural functioning and physiological functioning of neurons. It is essential for the development and maintenance of the central nervous system (CNS). There has been an increasing number of evidences which has reported that the lipid metabolism is highly involved in the pathological progression of neurodegenerative diseases, such as AD^85^ and PD.^86^ Outeiro et al. proved that α‐Syn aggregation induces abnormal accumulation of lipids.^87^ Mitochondrial dysfunction is an important reason for the onset of PD. The accumulation of intracellular lipids induces mitochondrial dysfunction and thus reduces the number of mitochondria, which further promotes lipid accumulation.^88^ Lipidomics is an emerging field that can provide new insights and new answers to improve early diagnosis and to track disease progression.^89^ It also plays an important role in the study of lipids in energy conversion and biofilm structure.^86^ Recently, a growing body of research suggests that disorders of lipid metabolism are strongly associated with PD.^90^, ^91^ Lipid metabolism is involved in the formation of a variety of biofilms in the cell.

### 3.1 Parkinson's disease and triacylglycerols

TAG is an ester, derived from three fatty acids and glycerol. Many studies have shown that the level of TAG in the blood of patients with PD is low when compared to healthy individuals.^92^, ^93^ High levels of TAG in the blood have been proven to have protective effects during PD.^94^, ^95^ Mice overexpressing α‐SynA53T cause decreased levels of TAG in PD mice model.^96^ Recently, more and more reports have highlighted that the level of intracellular TAG is significantly low in both PD rat model and patients with PD.^93^, ^97^ This association between TAG and PD risk could be linked to the level of DA. Rada et al. demonstrated that repeated intake of sugar increases the extracellular DA levels.^98^ High consumption of sugar can increase serum TAG.^99^

Faning et al. reported that TAG has a protective role against α‐Syn‐cytotoxicity, which is by inhibiting the accumulation of oleic acid and diglyceride (DG).^100^ Accumulation of diglyceride in the endoplasmic reticulum results in α‐Syn trafficking defects. High levels of oleic acid promote α‐Syn membrane binding, which enhances membrane‐associated toxicity.^100^ In other words, inhibition of TAG formation makes neurons more vulnerable to α‐Syn toxicity. A previous study had reported that mutation of the human gene ATP13A2 causes Parkinsonism with dementia.^101^ Marcos et al. have proven that overexpression of ATP13A2 in SH‐SY5Y cells reduces the levels of TAG.^102^ TAG is necessary for the synthesis of new membranes; therefore, it can be observed that probable overexpression of ATP13A2 disrupts the homeostasis of TAG, which causes PD.

**FIGURE 2:**
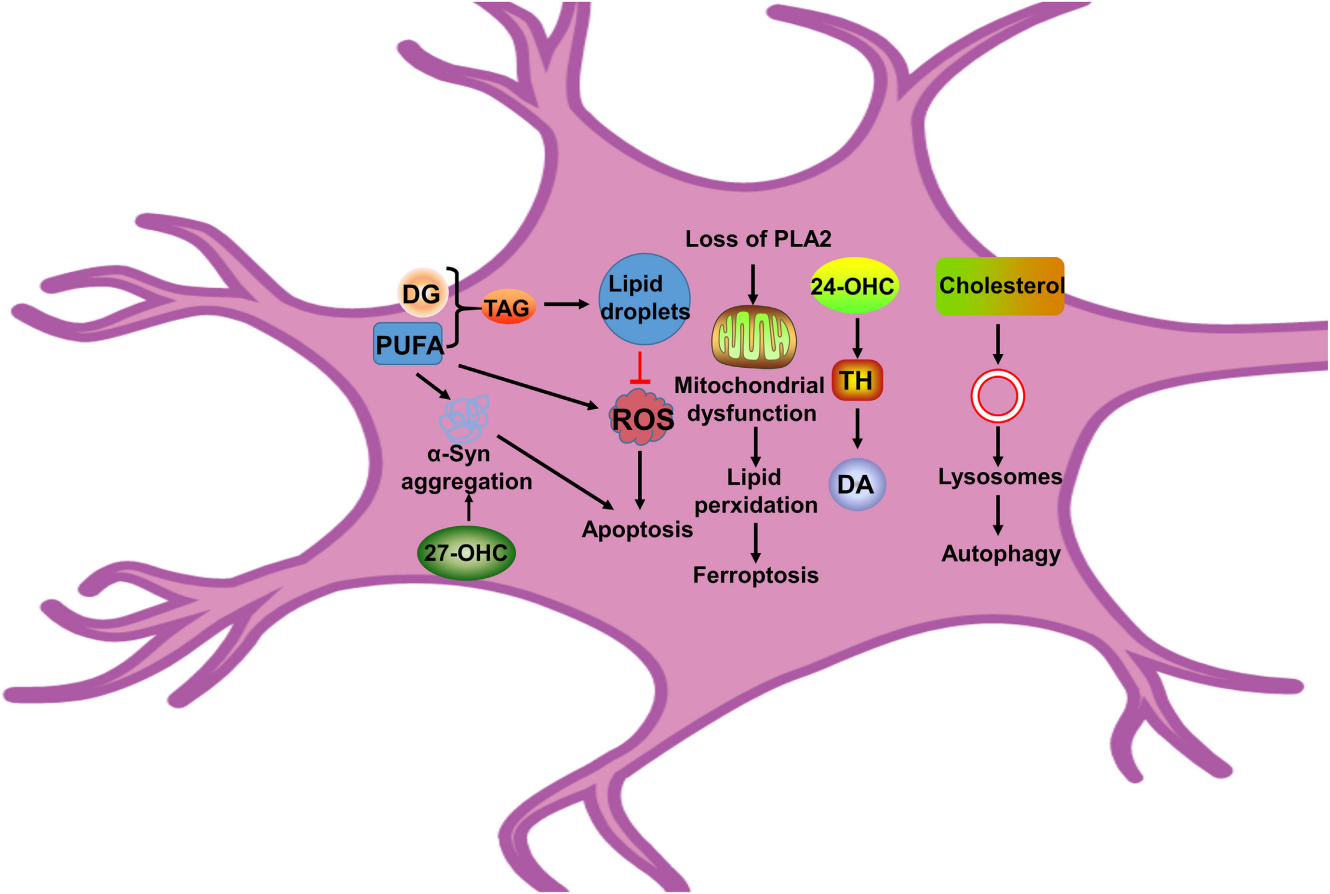
Overview of the lipid metabolism dysregulations in PD brain. Loss of PLA2 gene function results in lipid peroxidation and impaired mitochondrial function, which is associated with ferroptosis. 24‐OHC can activate tyrosine hydroxylase and promote dopamine synthesis. 27‐OHC can increase the level of α‐Syn, which induces apoptosis and eventually leads to neuronal death. The formation of lipid droplets can decrease the levels of ROS, which can inhibit apoptosis. Excess PUFA can increase the levels of α‐syn and ROS, which leads to apoptosis. PUFA forms TAG, which can be stored in lipid droplets. Inhibition of TAG formation makes neurons more vulnerable to α‐Syn toxicity. Disruption of cholesterol metabolism causes lysosomal dysfunction, which leads to autophagy dysfunction. PD, Parkinson's disease; PLA2, phospholipase A2; PUFA, polyunsaturated fatty acids; ROS, reactive oxygen species; TAG, triacylglycerol; α‐Syn: α‐synuclein.

Polyunsaturated fatty acids (PUFA) are an important component for the synaptic and mitochondrial membrane formations, and it is often susceptible to damage by reactive oxygen species (ROS). Previous studies have suggested that PUFA can promote the binding of α‐Syn and mitochondrial membrane, as well as promote the pathological aggregation of α‐Syn.^103^, ^104^, ^105^ Recent studies have reported that administration of omega‐3 PUFA is effective in alleviating the effects of PD.^106^ Interestingly, the levels of omega‐6 PUFA are higher in patients with PD compared with healthy control.^107^ Meng et al. found that administration of omega‐3 PUFA to mice model of PD successfully inhibited the neurotoxicity caused by MPP^+^, and omega‐6 PUFA is upregulated by the induction of MPP^+^.^108^ However, the specific molecular mechanism remains unclear. Campos et al. have shown that the esterification of arachidonic acid (a kind of omega‐6 PUFA) into TAG may cause a protective effect during Fe‐induced dopamine neuron injury.^109^ Therefore, with the above‐mentioned evidences, it can be suggested that excess PUFA forms TAG, which are stored in lipid droplets through esterification and prevents ROS damage to neurons caused by fatty acid β‐oxidation. This may explain the low levels of TAG in patients with PD. Excess PUFA generates ROS, which disrupts the membrane system, especially the cell membrane and mitochondria membrane. Mutation of α‐syn leads to an increase in TAG hydrolysis, resulting in a low level of TAG in cells.^110^, ^111^ This signifies an association between abnormal TAG metabolism and α‐syn aggregation. (Figure 2).

### 3.2 Parkinson's disease and cholesterols

Cholesterol plays a very important role in maintaining the integrity and fluidity of the cell membrane. It is mainly synthesized in the astrocytes through endoplasmic reticulum.^112^ It has a crucial role in regulating variety of physiological functions such as synthesis of various steroid hormones, vitamin D, and maintenance of neuronal development.^113^ Cholesterol is mainly distributed in lipid rafts, which are involved in a variety of signal regulation. Although the human brain makes up only about 2% of the body weight, it contains 25% of cholesterol and cholesterol derivatives.^114^, ^115^, ^116^ A previous study has reported that lipid rafts are strongly associated with PD‐related proteins such as α‐syn, LRRK2, Parkin, and DJ‐1.^117^ Cholesterol is present in synapses and can interact directly with neurotransmitter receptors. It is necessary for synaptic transmission and formation. Therefore, disturbances in cholesterol metabolism can cause changes in the functioning of brain neurons, leading to various neurological disorders.^118^, ^119^ An interesting phenomenon is seen when cholesterol synthesis in cells can produce lipoproteins which can be obtained through blood; however, in the brain, cholesterol must be synthesized de novo because lipoproteins cannot cross the blood–brain barrier.^114^, ^120^ Studies have suggested that changes in the level of cholesterol is closely associated with PD.^121^, ^122^

Cholesterol can be modified into various derivatives, that is, 24‐OHC (24‐hydroxy cholesterol), 25‐OHC (25‐hydroxy cholesterol), and 27‐OHC (27‐hydroxy cholesterol). 24‐OHC can activate tyrosine hydroxylase and promote dopamine synthesis.^116^ 27‐OHC can increase the level of α‐syn, which induces apoptosis and eventually leads to neuronal death.^123^ Studies have reported that hypercholesterolemia aggravates MPTP‐induced loss of dopaminergic neurons in the SN mice model of PD having motor dysfunction.^124^ At present, there are few reports on the role of cholesterol in PD.^124^ The specific mechanisms of cholesterol remain controversial.^125^ Eriksson et al. have proven that high levels of cholesterol have a dual effect on PD.^126^ The increased levels of cholesterol give rise to selective the permeability of lysosomal membranes and thus inhibit cell death while promoting the accumulation of α‐syn. The lysosomal function is strongly associated with PD.^127^ Studies have also proven that high levels of cholesterol may be a sign of immature or damaged lysosomes.^121^, ^128^ García‐Sanz et al. reported that the accumulation of lysosomal cholesterol alters the interaction between α‐syn and lipid rafts, promoting the oligomerization of α‐Syn. Eventually, these α‐Syn, which cannot be degraded by lysosomes, get fibrillated.^112^

Mutations in the Glucocerebrosidase (GBA) gene lead to an increased risk of PD. GBA knockout mice increase the levels of cholesterols.^121^ In addition, the high levels of cholesterols can promote the accumulation of autophagosomes, which can damage autophagy.^129^ Therefore, we suggest that the disruption of cholesterol metabolism causes lysosomal dysfunction, which leads to autophagy dysfunction. Eventually, it leads to neuronal death.

### 3.3 Parkinson's disease and phospholipids

Phospholipids are the main components of intracellular biofilms. It can be divided into two categories: glycerophospholipids and sphingolipids, which are the precursors for lipid mediators involved in signal transduction.^130^ Recent studies have reported that the accumulation of lipid peroxides can lead to membrane damage and thus induce cell death. Pan et al. reported that heterozygous deletion of synaptojanin1 (a phosphoinositide phosphatase) results in PD‐like symptoms in mice. It is caused by upregulating of phosphatidylinositol‐bis‐4,5‐phosphate (PI(4,5)P_2_). This may be because of the damage (PI(4,5)P_2_) causes the synaptic vesicle endocytosis, which results in the nerve terminals to selectively eliminate in the midbrain neurons.^131^, ^132^

Previous studies have identified that calcium‐independent phospholipase A2 (PLA2) is the causative gene for PD.^133^, ^134^, ^135^ Mori et al. mentioned that PLA2 is necessary for the maintenance and survival of dopaminergic neurons and for α‐Syn stability.^136^ In a mice model of PD, overexpression of the mutant A53T α‐Syn gene causes reduction in expression of PLA2, which leads to the accumulation of oxidized phospholipids and eventually ferroptosis.^137^ Loss of PLA2 gene function leads to lipid peroxidation and impaired mitochondrial function.^138^ Sánchez et al. suggested that downregulation of the gene PLA2G6 in the nervous system of zebrafish will result in the loss of dopaminergic neurons and parkinsonism.^139^ Therefore, lipid metabolism is one of the most important factors in maintaining normal neuronal function. Phospholipid peroxidation produced by disrupted phospholipid metabolism makes dopaminergic neurons susceptible to ferroptosis.^137^ Studies have revealed that ferroptosis is involved in PD. Lipid peroxide accumulation resulting in cell death is an important feature of ferroptosis. Hence, it is worth mentioning that ferroptosis induced by disorders in the phospholipid metabolism may be one of the causes of PD (Figure 2).

## 4 PARKINSON'S DISEASE AND AMINO ACIDS

The pathophysiology of PD is deeply associated with amino acid metabolism. Glutamate metabolism plays a significant role in the progress of PD. A metabolomics study has shown that alanine and phenylalanine levels in the cerebrospinal fluid are decreased in PD patients compared with normal subjects.^140^ Gliomas are known to release large amounts of glutamate through the cystine/glutamate antiporter system Xc‐.^141^ Glutamine is converted to glutamate and is taken into presynaptic terminals of glutamatergic neurons by excitatory amino acid transporters (EAATs). Increased extracellular glutamate concentration induces abnormal synaptic signaling leading to excitotoxicity and death of neurons.^142^

As a dopamine precursor, levodopa is used to increase dopaminergic neurotransmission in patients with PD. However, long‐term usage of levodopa leads to involuntary movements such as levodopa‐induced dyskinesia (LID) and overactivity of glutamatergic cortico‐striatal projections.^143^ Glutamate‐induced oxidative toxicity is closely associated with ferroptosis.^144^, ^145^ Many studies have reported that ferroptosis is involved in PD.^146^ Safinamides target the glutamatergic system selectively and reversibly by inhibiting monoamine oxidase‐B (MAO‐B). This restores the striatal dopaminergic tone and reduces the subthalamic/nigral glutamatergic hyperactivity through use‐dependent sodium channel blockade, which prevents calcium channel opening and results in the inhibition of abnormal glutamate release in a PD model.^147^, ^148^ Therefore, it can be mentioned that abnormalities in glutamate metabolism produce neurotoxicity and oxidative toxicity, which induce dopaminergic neuronal death.

Cysteine is one of the three amino acids that make up GSH. Studies have reported that the total contents of glutathione (GSH) and GSH/oxidized glutathione (GSSG) ratio is extremely low in the temporal cortex and cerebellum of patients with PD.^149^, ^150^, ^151^, ^152^, ^153^ This is accompanied by a higher susceptibility to oxidative stress. This finding suggests that the concentration of GSH, GSH/GSSG cycle, and genetic modifications in GSH homeostasis affects the ROS/reactive nitrogen species (RNS) of patients with PD.^151^ Low concentration of GSH results in oxidative stress and consequently may induce mitochondrial dysfunction, oxidative damage in DNA, and proteins, and triggers neurodegeneration resulting in PD.^154^, ^155^ GSH depletion can cause an accumulation of extracellular glutamate in astrocyte cultures.^156^ Reduced cysteine uptake leads to a reduction in GSH, which is associated with glutamate neurotoxicity.^157^, ^158^ In addition, glutathione peroxidase 4 (GPX4) depends on GSH a key enzyme of ferroptosis.^159^ All these findings indicate that the activity and concentration of cysteine are involved in PD. This involvement is seen during regulation of the redox homeostasis and bioenergetic metabolism.

It has been reported that the level of γ‐aminobutyric acid (GABA), an inhibitory neurotransmitter, is low in the cerebrospinal fluid of patients with PD.^160^ It is also involved in the occurrence and development of PD.^161^ GABA can control the activity of DA neurons of the SN. Loss of GABA or its synthesizing enzyme glutamic acid decarboxylase (GAD) has been observed in patients with PD.^162^ Kuruvilla et al. reported that combination of GABA, serotonin, and autologous bone marrow cells can inhibit 6‐hydroxydopamine‐induced (6‐OHDA) PD.^163^ Glycine is another inhibitory neurotransmitter that acts as a co‐agonist with glutamate at the site of glutamate receptors.^164^ Inhibition of glycine transport can promote the function of dopamine axons.^165^ Therefore, controlling the metabolism of GABA and glycine may be a potential target for the treatment of PD.

## 5 DISCUSSION

In this review, we summarize the relationship between different energy metabolisms (glucose metabolism, lipid metabolism, and amino acid metabolism) with PD (Figures 1 and 2). Besides, we also summarize the key finding of metabolic pathway (Table 1). Sugar is the main energy supplier for the human body. Patients with PD prefer to consume more sugar than normal people.^166^ This is because an increase in sugar consumption increases the level of dopamine in the brain, which may be a compensatory mechanism in patients with PD.^166^, ^167^, ^168^ Recent studies have proven that diabetes can increase the risk of PD. Many drugs used for targeting glucose metabolism, such as terazosin, meclizine, and alfuzosin, have been identified as possible treatment of options for PD (Table 2). However, the potential role of GLUTs and PPP remains unmapped. Promoting glycolysis gluconeogenesis contributes to alleviating PD. Through recent findings, it has been shown that disruptions in glucose metabolism by regulating the concerned pathways eventually lead to alterations in ATP and ROS levels. Therefore, improving these intracellular pathways associated with apoptosis, autophagy, and ferroptosis by regulating the balance of energy metabolism may be an innovative method to treat PD. Previous studies have proven that astrocyte‐neuron lactate shuttle (ANLS) can consume sugar through anaerobic glycolysis and provide energy metabolic support to neurons.^169^, ^170^, ^171^ Neurons then produce ATP through oxidative phosphorylation. Therefore, there must be a relationship between neurons and astrocytes in the relevant pathways of sugar metabolism or their intermediate products by regulating the accumulation of ROS and thus inducing PD. It is worth to investigating whether astrocytes or/and neurons play any role in dopamine neuron damage. For example, while studying about the relationship between PD and sugar transport, more focus needs to be diverted toward astrocytes. This is because, in the brain, glycogen is mainly stored in the astrocytes. Jia et al. demonstrated that neuronal metabolism of excess lactate leads to more mitochondrial reactive oxygen species (mtROS) production.^172^

**TABLE 1:** Summary of key findings in each section of metabolic pathway.

Metabolic pathway	Study	Key finding	Reference
Glucose transporter	MPTP‐induced mouse model of PD	GLUT1 is decreased in the striatum	31, 34
	MPP^+^‐induced N27 cell model of PD	Upregulating the expression of GLUT3 can inhibit the neurotoxicity induced by MPP^+^	35
Glycolysis	Human	PGK‐1 mutations contribute to vulnerability to parkinsonism in humans	45, 46
	6‐OHDA‐induced rat model of PD	Upregulation of leptin induced by NaHS can promote glycolysis and alleviate PD	48
	Rotenone‐induced SH‐SY5Y cell model of PD; rotenone/MPTP‐induced mouse model of PD	Overexpression of HK2 can alleviate the symptoms of PD by promoting glycolysis	49, 50
OXPHOS	Human; Human	mtDNA mutations in SN neurons of patients with PD and aged humans are accompanied by a deficiency in OXPHOS	60, 61
Gluconeogenesis	MPP^+^‐induced N2A cell model of PD	Intermediates of gluconeogenesis have a neuroprotective effect against MPP^+^‐induced neurotoxicity	71
	MPTP‐induced mouse model of PD	Gluconeogenesis‐related proteins are involved in PD	72
PPP	MPTP‐induced mouse model of PD; Human; Human	G6PD plays an important role in PD, however, the role of G6PD in PD is still controversial	75, 79, 80
TAG	Human; Human	The level of TAG in the blood of patients with PD is low, when compared to healthy individuals	92, 93
	Human; Human	High level of TAG in the blood has protective effects in PD	94, 95
	MPP^+^‐induced mouse model of PD	Administration of omega‐3 PUFA is effective in alleviating the effects of PD	108
Cholesterol	SH‐SY5Y cells	24‐OHC can activate tyrosine hydroxylase and promote dopamine synthesis	116
	SH‐SY5Y cells	27‐OHC can increase the level of α‐syn, which induces apoptosis and eventually leads to neuronal death	123
	MPP^+^‐induced BE(2)‐M17 cell model of PD	High level of cholesterol has a dual effect on PD	126
Phospholipids	iPLA2‐VIA‐deficient Drosophila	PLA2 is necessary for the maintenance and survival of dopaminergic neurons and for α‐Syn stability	136

Abbreviations: 24‐OHC, 24‐hydroxy cholesterol; 27‐OHC, 27‐hydroxy cholesterol; 6‐OHDA, 6‐hydroxydopamine; G6PD, glucose‐6‐phosphate dehydrogenase; GLUT, glucose transporter; HK2, hexokinase 2; MPP^+^, 1‐methyl‐4‐phenylpyridinium; MPTP, 1‐Methyl‐4‐phenyl‐1,2,3,6‐tetrahydropyridine; OXPHOS, oxidative phosphorylation; PGK‐1, phosphoglycerate kinase 1; PLA2, calcium‐independent phospholipase A2; PPP, pentose phosphate pathway; PUFA, Polyunsaturated fatty acids; SN, substantia nigra; TAG, triacylglycerols.

**TABLE 2:** Drugs targeting glucose metabolism for the potential treatment of Parkinson's disease.

Drug	Study	Target	Pathway	Reference
Terazosin	MPTP‐induced mouse model of PD; 6‐OHDA‐induced rat model of PD; rotenone‐induced fly model of PD	PGK‐1	Glycolysis	43
Meclizine	6‐OHDA‐induced SH‐SY5Y cell model of PD	PFK	Glycolysis	51
Alfuzosin	MPTP‐induced mouse model of PD; a decreased risk of developing PD in human	PGK‐1	Glycolysis	43, 184
Doxazosin	MPTP‐induced mouse model of PD; a decreased risk of developing PD in human	PGK‐1	Glycolysis	43, 184
6‐aminonicotinamide	Paraquat‐induced SK‐N‐SH cell model of PD	G6PD	PPP	78
Hydrazine sulfate	MPP^+^‐induced N2A cell model of PD	PEPCK	Gluconeogenesis	71
STF‐31	Paraquat‐induced N27 cell model of PD	GLUT	Glucose transport	36

Abbreviations: PEPCK, Phosphoenolpyruvate carboxykinase; MPP^+^, 1‐methyl‐4‐phenylpyridinium; PGK‐1, phosphoglycerate kinase 1; 6‐OHDA, 6‐hydroxydopamine; PFK, phosphofructokinase; G6PD, glucose‐6‐phosphate dehydrogenase; PPP, pentose phosphate pathway; GLUT, glucose transporter; MPTP, 1‐Methyl‐4‐phenyl‐1,2,3,6‐tetrahydropyridine; STF‐31, 4‐[[[[4‐(1,1‐dimethylethyl)phenyl]sulfonyl]amino]methyl]‐N‐3‐pyridinyl‐benzamide.

Neurons are highly susceptible to oxidative damage because of their high unsaturated fatty acid content, high oxygen consumption, and relatively weak antioxidant defense mechanisms. The formation of TAG not only protects the neurons from α‐Syn, but also inhibits fatty acid accumulation thereby preventing β‐oxidation. Excessive production of ROS causes disruption of mitochondrial function as well as cell membrane integrity, which induces apoptosis or ferroptosis. Hantikainen et al. demonstrated that high consumption of saturated fats may increase the risk of PD, and a diet low in saturated fat may be beneficial in preventing PD.^173^ It is known that cholesterol plays a key role in maintaining the integrity and fluidity of cell membrane. Therefore, we suggest that abnormalities in cholesterol metabolism may affect cellular autophagy and thus neurological function.

It is worthwhile to propose that administration of omega‐3 PUFA helps to alleviate PD. Eating more omega‐3 PUFA than other fats may be beneficial for patients with PD. It is certain that abnormalities in lipid metabolism can increase the chances of PD.^174^, ^175^, ^176^ Food therapy has a good chance of improving PD. A variety of amino acid neurotransmitters, such as glutamate, GABA, and glycine, is said to have its effect on PD. Neurons use a lot of energy to release neurotransmitters; however, excessive excitation of neurons can cause neurotoxicity. Therefore, the balance of amino acid neurotransmitter metabolism is essential in order to maintain a perfect neuronal functioning. Magistretti et al. reported that glutamate can stimulate astrocytes to utilize glucose and release lactic acid (LA) through glycolysis. When energy in the cell is insufficient, the cell produces energy by breaking down amino acids. We can conclude that abnormality of amino acid metabolism affects PD by regulating neuronal excitability.

In summary, abnormalities in energy metabolism are mainly involved in the pathological process of PD by disrupting mitochondrial dysfunction or damaging membrane integrity in neurons. Inhibiting the accumulation of intracellular ROS is an important approach to alleviate PD. Since the functioning of various enzymes can effectively control intracellular energy metabolism through glucose metabolism and lipid metabolism, inhibitors or activators of related enzymes have potential roles in alleviating PD. This study points out that oxidative stress may be the underlying cause of induction for neuronal death, which may be associated with apoptosis, autophagy, and ferroptosis.

## 6 CONCLUSION

PD patients exhibit disturbances in glycolysis and oxidative phosphorylation, decreased triglyceride levels, disrupted phospholipid metabolism, loss of GABA neurons, and other changes in energy metabolism. Disturbances in metabolism affect the progression of PD by triggering autophagy, apoptosis, and ferroptosis. Patients with multiple metabolic disorders like diabetes are at high risk of developing PD.

Increasing evidences have reported that the metabolism‐related indicators are associated with the occurrence and progression of PD. For example, disturbances in glucose metabolism in the SN can be a marker for the diagnosis of PD.^177^ Xicoy et al. showed that the genetic overlap between the specific lipids in the blood and PD can be identified as a novel diagnostic biomarker in PD.^178^ PD is related to the abnormal lipid metabolism according to integrated proteomics and metabolomics analysis which indicates that improving lipid metabolism is a promising method for the treatment of PD.^179^ Also, abnormalities in amino acid metabolism have also been widely noted in PD such as glycine, glutamate and GABA.^1^, ^180^ Heilman et al. reported that the kynurenine metabolites may be a biomarker for PD and/or involved in the pathogenesis of PD.^181^ Zhang et al. showed that modulation of glutamine metabolism has a potential therapeutic effect for the treatment of PD.^182^ A recent study has shown that upregulation of serine levels is a biochemical signature of dopaminergic neuronal degeneration in patients with PD.^183^ Therefore, it can be concluded that metabolism‐related indicators may be used as markers for monitoring PD progression. Drugs related to targeted metabolic pathways may have a potential role in the treatment of PD. Furthermore, since the role of PPP and cholesterol with PD is not well understood, it is worthwhile to investigate the potential role of PPP and cholesterol in the treatment of PD. In the future, we hope to improve the life of PD patients through research on energy metabolism.

## AUTHOR CONTRIBUTIONS

The authors declare no competing financial interests. Xiang Li and Yan Chen were responsible for the study concept and design. Hangzhen Li, Fancai Zeng, Cancan Huang, and Qiqi Pu drafted the manuscript. Xiang Li and Elizabeth Rosalind Thomas provided a critical revision of the manuscript for important intellectual content. All authors read and approved the final version.

## FUNDING INFORMATION

This study was financially supported by grants from the Science and Technology Strategic Cooperation Project of the Luzhou People's Government and Southwest Medical University (No. 2019LZXNYDJ34) and the undergraduate innovation and entrepreneurship training program (S202110632241). This study was financially supported by Sichuan Science and Technology Program (Grant No. 2022YFS0623 and 2023JDRC0109).

## CONFLICT OF INTEREST STATEMENT

The authors declare no competing financial interests.

## Data Availability

The data that support the findings of this study are openly available in The National Center for Biotechnology Information at https://www.ncbi.nlm.nih.gov/.
